# A Retrospective Study of Herpetic Keratitis in Patients with Keratoconus after Crosslinking Surgery

**DOI:** 10.3390/jcm10122684

**Published:** 2021-06-18

**Authors:** Ewa Wróblewska-Czajka, Anna Nowińska, Dariusz Dobrowolski, Dominika Szkodny, Edward Wylęgała

**Affiliations:** 1 Chair and Clinical Department of Ophthalmology, Faculty of Medical Sciences in Zabrze, Medical University of Silesia, 40-760 Katowice, Poland; atrum2@gmail.com (A.N.); dominikacholewa1@gmail.com (D.S.); wylegala@gmail.com (E.W.); 2 Department of Ophthalmology, District Railway Hospital in Katowice, 40-760 Katowice, Poland; dardobmd@wp.pl

**Keywords:** herpetic keratitis, cross-linking, keratoconus

## Abstract

Background: The aim of this study was to perform a retrospective analysis of patients who underwent cross-linking for keratoconus, in the Department of Ophthalmology of the Medical University of Silesia in Katowice, between 2011 and 2020, regarding the occurrence of herpetic keratitis after the procedure. Methods: We analyzed the medical history of 543 patients who underwent cross-linking surgery. Results: In the analyzed group, there were nine cases of herpetic keratitis (six men and three women), aged from 16 to 40 years (mean 26.2 years). The mean follow-up period was 49.3 months (16–82 months). The average time from surgery to the manifestation of the first symptoms of keratitis was 4.3 days (2–6 days). In two cases, iritis was observed, and in one of them, iritis was the first symptom. After systemic and topical administration of acyclovir, ulceration healed in all patients. Corneal healing time ranged from 10 days to 3 weeks (average 13.7 days). In one patient, a recurrence of the inflammation was observed after 8 months. Conclusion: Patients should be carefully observed in the early post-CXL period. Herpetic keratitis could be induced by CXL even in patients with no history of herpetic disease.

## 1. Introduction

The corneal cross-linking (CXL) procedure was first performed over 20 years ago [1]. Since then, it has been successfully used primarily to stop the progression of corneal ectasia, as well as in the cases of bullous keratopathy, sterile corneal melting, and infectious keratitis of bacterial and fungal origin [2,3,4]. CXL involves the photopolymerization of stromal fibers by the combined action of a photosensitizing substance (riboflavin) and UVA radiation. The procedure is generally considered safe. However, it is not free from complications. The most frequent complications associated with CXL are transient or persistent corneal haze, epithelial disorders, infections, peripheral sterile infiltrates, stromal melting, endothelial damage, and herpes simplex virus (HSV) activation [5,6]. Herpetic keratitis is one of the symptoms of HSV reactivation. During the initial infection, the virus multiplies within the epithelial cells and then from the sensory nerves it travels to the sensory ganglion and remains there in the dormant form. The reasons for HSV reactivation are not entirely clear. Some authors suggest that factors such as exposure to sunlight, stress, menstruation, surgery, or fever are associated with virus reactivation, but few papers deny this relationship. Secondary viral replication within the epithelial cells results in dendritic or geographic keratitis. The immunological response to the virus antigen leads to the development of stromal keratitis. Recurrent infections cause the loss of ganglion cells, leading to a decrease in corneal sensation and consequently resulting in trophic corneal ulcer. HSV infection can also lead to uveitis, trabeculitis, and retinitis [7]. It is not a commonly described complication after cross-linking surgery. Nevertheless, because of its serious consequences, it is important to determine the probability of occurrence of herpetic keratitis. The aim of this study was to perform a retrospective analysis of patients who underwent cross-linking for keratoconus, in the Department of Ophthalmology of the Medical University of Silesia in Katowice, between 2011 and 2020, with regard to the occurrence of herpetic keratitis as a complication after the procedure.

## 2. Materials and Methods

This study was performed in accordance with the Declaration of Helsinki. We analyzed the medical history of 543 patients who underwent cross-linking surgery for progressive keratoconus. The patients included 312 males and 231 females, at a mean age of 22.7 years (14–47 years). The mean follow-up period was 6.7 years (1–9 years). All patients received the same treatment, which was performed according to the Dresden protocol (an epi-off protocol). After the treatment, drops of levofloxacin (5 mg/mL) and dexamethasone (1 mg/mL) were applied and a soft bandage contact lens (monthly, Air Optix Aqua; Ciba Vision, 14.2 diameter, 8.6 base curve) was placed. The patients were instructed to use levofloxacin and dexamethasone drops and artificial tears five times a day. When herpetic keratitis was found, we analyzed the previous HSV history of patients, the time between crosslinking and herpetic keratitis, HSV infection symptoms, time of complete healing of ulcer, and follow-up time.

## 3. Results

In the analyzed group, there were nine cases of herpetic keratitis, including six men and three women aged 16–40 years (mean 26.2 years). All patients came to the hospital before the scheduled first visit 7 days after surgery complaining of pain, photophobia, and irritation symptoms in the treated eye. The average time from surgery to the emergence of symptoms was 4.3 days (2–6 days). Five patients showed a dendritic ulcer on slit-lamp examination, and two showed geographic ulceration. In the other two patients, who reported on the second and third day after the cross-linking treatment, ocular irritation predominated, and dendritic ulceration appeared after the three next days. Signs of iritis were found in two cases.

**Figure 1:**
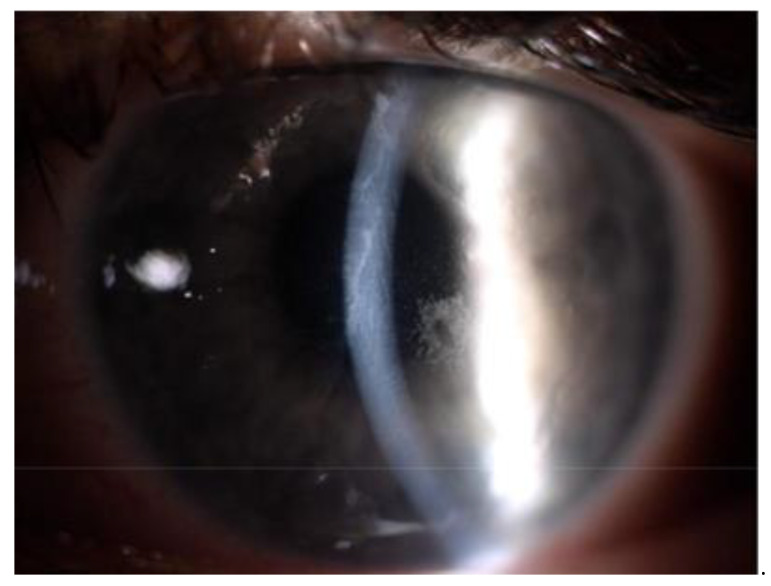
Slit lamp image of the left eye of a patient aged 16 years two days after cxl surgery for progressive keratoconus. Visible postoperative epithelial abrasion, edema, and haze of the corneal and numerous small deposits on the endothelium.

**Figure 2:**
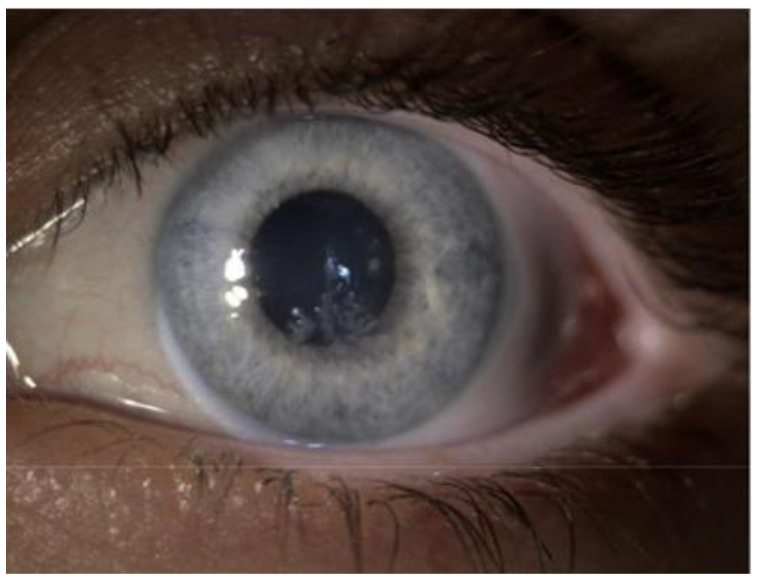
Slit lamp image of the same patient’s eye 4 days after cxl. Visible cornea covered with epithelium with dendritic ulceration paracentral. Reduction in endothelial deposits and significant reduction in corneal edema and haze.

**Figure 3:**
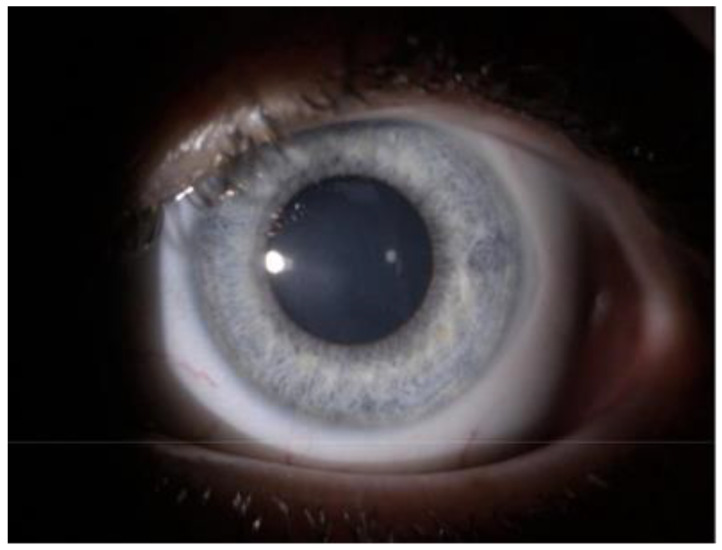
Slit lamp image of the left eye of the same patient 2 months after cxl.

After HSV diagnosis, the treatment was modified. Except for one patient with significant iritis, corticosteroids were discontinued in the other patients who underwent treatment. Topical acyclovir (Viru-POS 30 mg/g, 4.5 g Ursapharm, Poland) was administered five times a day until the ulcer was healed and then three times a day for a week. Oral acyclovir 400 mg was administrated five times daily until the ulcer completely healed and then two times for a further 2 weeks. Steroids were continued again after complete epithelial defect healing. Corneal healing time ranged from 10 days to 3 weeks (average 13.7 days). In two cases, corneal haze was observed in the healed ulcer for about 2 weeks (Figure 1, Figure 2 and Figure 3).

During the follow-up period, only one patient experienced a recurrence of herpetic keratitis after 8 months. However, the symptoms resolved after 10 days of antiviral treatment without additional complications. The detailed description of the series is presented in Table 1.

## 4. Discussion

The mechanism of the reactivation of HSV after CXL may be related to epithelial abrasion, UV exposure, use of topical steroid drops, and stress [8]. Although this complication has already been reported in the literature [8,9,10], to our knowledge this study is one of the few to analyze such a large group of patients for HSV infection, and thus allowed to determine the risk of this potentially serious complication. It is also worth mentioning that bilateral herpetic keratitis can occur after simultaneous cross-linking in both eyes [11]. In most of the available articles, the patients did not have a previous positive medical history of HSV infection [7,9]. This may be due to the fact that the primary HSV infection is usually asymptomatic or scarcely symptomatic and may be overlooked. Moreover, in the group described by us, only one patient had herpes labialis. Interestingly, this patient was advised to take acyclovir 400 mg orally five times daily for 3 days prior to surgery as prophylaxis, but despite this he developed inflammation and the ulcer healing period was found to be one of the longest. Other studies have reported that reactivation of inflammation occurred most often between the fourth and ninth day after surgery [7,8,9,10]. Usually, the first control is carried out after 7 days of surgery because after this time the contact lens can be already removed, and the use of antibiotic drops can be stopped. In this study, all our patients had symptoms before their first visit. Therefore, patients should be advised to seek immediate medical attention if they experienced pain, eye irritation, or photophobia. In our series, symptoms appeared, on average, on day 4 after the CXL procedure. The earliest symptoms appeared in the youngest patient, a 16-year-old girl. After 2 days of treatment, the patient had eye irritation, significant corneal edema, and iritis. After the next 3 days, corneal dendritic inflammation appeared with corneal re-epithelialization. It should be noted that if virus activation occurs before re-epithelialization and after epithelial abrasion during surgery, we may not have a typical picture of dendritic keratitis. Viral infection may be suggested by eye irritation, signs of iritis, and prolonged or absent epithelialization. Due to the use of steroid drops after the procedure, the inflammation may take the form of geographical ulceration. Interestingly, some reports show the beneficial effects of cross-linking in patients with herpetic keratitis [12]. In this study, in the two described cases with confirmed herpetic inflammation, corneal ulceration healed after the cross-linking procedure. This suggests that the procedure probably had a reducing effect on neurotrophic corneal melting occurring after herpetic inflammation [13].

## 5. Conclusions

Patients should be carefully observed in the early post-CXL period. Herpetic keratitis could be induced by CXL even in those with no history of herpetic disease.

## Figures and Tables

**Table 1:** The detailed description of the series.

Patient	1	2	3	4	5	6	7	8	9
Age	38	40	31	16	21	18	24	19	29
Gender	M	M	F	F	M	M	M	F	M
Keratoconus degree (Amsler–Krumeich classification)	2–3	2	3	3	1–2	1–2	2	2	2–3
Previous HSV history	No	Recurrent herpes simplex labialis	No	No	No	No	No	No	No
Time of CXL to HSV reactivation (days)	4	6	4	2	5	4	5	3	6
HSV infection symptom	Dendritic ulcer, Transient corneal opacification	Dendritic ulcer	Dendritic ulcer, Transient corneal opacification	Dendritic ulcer, Significant iritis, Significant corneal edema	Dendritic ulcer	Geographic ulcer	Dendritic ulcer	Dendritic ulcer Mild iritis	Geographic ulcer
Time of complete ulcer healing (days)	10	18	20	21	11	14	10	14	14
Follow-up time (months)	38	20	82	36	16	79	52	58	63
Recurrence of HSV keratitis in follow-up time	No	No	No	No	No	No	No	Yes, one time after 8 months	No
Kmean before CXL	43.6	42.5	45.8	45.5	40.3	42.0	48.2	43.6	47.8
Kmean 1 year after CXL	43.2	41.8	45.8	48.4	40.2	41.9	48.0	43.6	47.8
Kmax before CXL	53.6	50.2	54.4	56.5	45.4	49.2	54.0	50.6	58.6
Kmax 1 year after CXL	52.0	49.0	54.0	57.4	47.0	48.9	53.2	49.7	57.0

## Data Availability

Data available on request due to restrictions e.g., privacy or ethical.

## References

[1] Spörl E., Seiler T., Huhle M., Kasper M. (1997). Increased rigidity of the cornea caused by intrastromal cross-linking. Ophthal Mologe.

[2] Ting D.S.J., Henein C., Said D.G., Dua H.S. (2019). Photoactivated chromophore for infectious keratitis—Corneal cross-linking (PACK-CXL): A systematic review and meta-analysis. Ocul. Surf..

[3] Choy B.N.K., Ng A.L.K., Zhu M.M., Liu C.C., Xu S., Lai J.S.M. (2020). Randomized Control Trial on the Effectiveness of Collagen Cross-linking on Bullous Keratopathy. Cornea.

[4] Abbouda A., Abicca I., Alió J.L. (2018). Current and Future Applications of Photoactivated Chromophore for Keratitis-Corneal Collagen Cross-Linking (PACK-CXL): An Overview of the Different Treatments Proposed. Semin. Ophthalmol..

[5] Abbouda A., Abicca I., Alió J.L. (2016). Infectious Keratitis Following Corneal Crosslinking: A Systematic Review of Reported Cases: Management, Visual Outcome, and Treatment Proposed. Semin. Ophthalmol..

[6] Dhawan S., Rao K., Natrajan S. (2011). Complications of corneal collagen cross-linking. J. Ophthalmol..

[7] Al-Dujaili L.J., Clerkin P.P., Clement C., McFerrin H.E., Bhattacharjee P.S., Varnell E.D., Kaufman H.E., Hill J.M. (2011). Ocular herpes simplex virus: How are latency, reactivation, recurrent disease and therapy interrelated?. Future Microbiol..

[8] Kymionis G.D., Portaliou D.M., Bouzoukis D.I., Suh L.H., Pallikaris A.I., Markomanolakis M., Yoo S.H. (2007). Herpetic keratitis with iritis after corneal crosslinking with riboflavin and ultraviolet A for keratoconus. J. Cataract. Refract. Surg..

[9] Al-Qarni A., AlHarbi M. (2015). Herpetic Keratitis after Corneal Collagen Cross-Linking with Riboflavin and Ultraviolet-A for Kerato-conus. Middle East Afr. J. Ophthalmol..

[10] MYuksel N., Bilgihan K., Hondur A.M. (2011). Herpetic keratitis after corneal collagen cross-linking with riboflavin and ultraviolet-A for progressive keratoconus. Int. Ophthalmol..

[11] Sitaula S., Singh S.K., Gurung A. (2019). Bilateral viral keratitis following corneal collagen crosslinking for progressive keratoconus. J. Ophthalmic Inflamm. Infect..

[12] Khalili M.R., Jahadi H.R., Karimi M., Yasemi M. (2017). Corneal Collagen Cross-linking for Treatment of Bacterial and Herpetic Keratitis. J. Clin. Diagn. Res..

[13] Xu X., Liu T., Li H. (2018). Effect of Collagen Cross-Linking on Alkali Burn-Induced Corneal Neovascularization in Rabbits. J. Ophthalmol..

